# Determinants of delay in malaria treatment-seeking behaviour for under-five children in south-west Ethiopia: a case control study

**DOI:** 10.1186/1475-2875-9-320

**Published:** 2010-11-11

**Authors:** Alemayehu Getahun, Kebede Deribe, Amare Deribew

**Affiliations:** 1 Wollega University, Nakamte, Ethiopia; 2 Fayya Development Association, Addis Ababa, Ethiopia; 3 Department of Epidemiology, Director of publication and Extension, Jimma University, Jimma, Ethiopia

## Abstract

**Background:**

Prompt diagnosis and timely treatment of malaria within 24 hours after onset of first symptoms can reduce illness progression to severe stages and therefore, decrease mortality. The reason why mothers/caretakers delay in malaria diagnosis and treatment for under-five children is not well studied in Ethiopia. The objective of this study was to assess determinants of malaria treatment delay in under-five children in three districts of south-west Ethiopia.

**Methods:**

A case control study was conducted from March 15 to April 20, 2010. Cases were under-five children who had clinical malaria and sought treatment after 24 hours of developing sign and symptom, and controls were under-five children who had clinical malaria and sought treatment within 24 hours of developing sign and symptom of malaria. Data were collected by trained enumerators using structured questionnaire. Data were entered in to Epi Info version 6.04 and analyzed using SPSS version 16.0. To identify determinants, multiple logistic regression was done.

**Results:**

A total of 155 mothers of cases and 155 mothers of controls were interviewed. Mothers of children who were in a monogamous marriage (OR = 3.41, 95% CI: 1.39, 8.34), who complained about the side effects of anti-malarial drugs (OR = 4.96, 95% CI: 1.21, 20.36), who had no history of child death (OR = 3.50, 95% CI: 1.82, 6.42) and who complained about the higher cost of transportation to reach the health institutions (OR = 2.01, 95% CI: 1.17, 3.45) were more likely to be late for the treatment of malaria in under-five children.

**Conclusion:**

Effective malaria control programmes should address reducing delayed presentation of children for treatment. Efforts to reduce delay should address transport cost, decentralization of services and increasing awareness of the community on early diagnosis and treatment.

## Background

By the mid-20th century, malaria was eliminated as a major health problem in many part of the world. In many parts of sub-Saharan Africa, it is still the largest contributor to the burden of disease and premature death [1-3].

During the past decades, numerous large-scale initiatives have been undertaken with the goal of reducing or eradicating the burden of malaria in the developing world. However, the ambitious goals set by these programmes for reducing the burden of malaria in the near future appear unlikely to be met [4]. Mortality from malaria is the major burden in under-five children. Most deaths occur at the community level, outside the health institution. Effective treatment exists, but it must be administered promptly and timely by trained personnel in order to be effective [5,6].

In Africa, an estimated 300-500 million cases of malaria occur each year resulting in approximately one million deaths. Among death due to malaria occurring in Africa more than 90% are in under-five children that results in brain damage [7-9]. However, it is generally accepted that most malaria deaths can be prevented when clinical cases are promptly diagnosed and effectively treated. Major factors affecting the outcome of the diseases are health-seeking behaviour and socio-economic status, which determine access to health services [2].

In young children, malaria can progress from a mild to severe case within 24 hours after the onset of symptoms. Prompt diagnosis and timely malaria treatment within 24 hours after onset of first symptoms can reduce illness progression to severe stages and, therefore, decrease mortality [10,11]. In Ethiopia, acute febrile illnesses are the leading causes of morbidity and mortality among under-five children [12-14]. Over five million episodes of malaria are estimated to occur annually and it is the top leading cause of outpatient visit and inpatient mortality [15,16].

The Ethiopian malaria control and prevention strategy gives due emphasis for early diagnosis and prompt treatment [17]. However, children with malaria often times seek treatment in the late stage of the disease. Information concerning determinants of malaria treatment delay in under-five children is insufficient in Ethiopia. Therefore, this study could contribute to understanding determinants of malaria treatment delay for under-five children in local context, which is essential for malaria control in children.

Malaria is a major public health problem in Ethiopia; it contributes up to 20% of under-five deaths. Tragically, in epidemic years, mortality rates of nearly 100,000 children are not uncommon. In the last major malaria epidemic in 2003, there were up to 16 million Malaria is a major public health problem in Ethiopia; it contributes up to 20% of under-five deaths. Tragically, in epidemic years, mortality rates of nearly 100,000 children are not uncommon. In the last major malaria epidemic in 2003, there were up to 16 million.

## Methods

### Study area and period

A case control study was conducted from March 15 to April 20, 2010 in six health centres (Nada, Asandabo, Dimtu, Akko, Sokoru and Deneba Health centres) located in Omo Nada, Tiro Afeta and Sokoru Districts in south-west Ethiopia. The three Districts were selected purposively based on their malaria endemicity and all health centres in selected Districts were included. The study health centres are found around the Gilgel Gibe Hydroelectric dam where malaria is known to be endemic [18,19].

### Study population

The study population consisted of cases and controls. Cases were under-five children who had one or more signs/symptoms of malaria and positive for plasmodium species based on blood film examination and sought treatment in the health centres after 24 hours of developing sign and symptoms. Controls were under-five children who had one or more signs/symptoms of malaria and positive for *Plasmodium *species based on blood film examination and sought treatment within 24 hours of developing sign and symptoms at the health centres.

### Sample size and sampling procedure

The sample size was calculated using Epi-Info version 6.04 statistical software. The sample size was calculated by taking distance from health institutions as a main exposure variable. From literature, it was found that proportion of individuals travelling a distance of three kilometres or more to reach the nearby health centre among controls was 27.65% [20]. By considering 80% power, Odds ratio of 2.0, and case to control ratio of 1:1, the total sample size was 310 children (155 cases and 155 controls).

The survey was conducted in under-five clinics to identify and register cases and controls. Cases and controls who had malaria in the health centres were included after blood film examination. Children who had fever and negative for *Plasmodium falciparum *were excluded. Children visiting the health centres during the study period and fulfilling inclusion criteria were recruited till the sample size was achieved. For a case found during data collection, a consecutive control was recruited from the same health centre.

The study excluded private facilities since majorities of the private health facilities did not have laboratory facilities and treat malaria empirically based on signs and symptoms.

### Data collection method and tools

Cases and controls were interviewed by trained grade 10 completed students using pre-tested and structured questionnaire. The survey tool was initially prepared in English, and translated to Afan Oromo (local language), and checked for its consistency through back translation to English by three different individuals who were health professionals. The questionnaire had two parts. The first part (socio-demographic section) encompasses age, sex, occupation, educational status, income, ethnicity, marital status, family size and communication materials. In the second part, behavioural factors, such as perception and knowledge of mothers, and health system and infrastructure-related factors were assessed.

To assess the knowledge of the respondents, 10 questions related to the signs and symptoms, mode of transmission, prevention and treatment of malaria were asked. For each question, a correct answer was given a score 1 and an incorrect answer a score 0. A total knowledge score was constructed by summing all the scores of the knowledge questions. Individuals who scored above the mean were categorized as knowledgeable. Diagnosis of malaria was made by thick and thin blood films prepared from a finger prick and stained with Giemsa in health centres. Each slide was read by experienced laboratory technicians. Absence of malaria parasite in 200 high power ocular fields of the thick film was considered as negative [11].

### Data analysis

Data were first checked manually for completeness and then entered into Epi-Info version 6.04. After data entry and cleaning, the data was transferred to SPSS version 16.0 for analysis. Bivariate analysis between dependent and independent variables was performed using binary logistic regression. To control the effect of confounding variables, multivariate logistic regressions were done. Variables which showed significant association in the bivariate analyses (P < 0.05) were candidates for the multivariate stepwise logistic regression model. Variables which were not statistically significant in the bivariate analysis were excluded in the multiple logistic regression model since they have small measure of effect (Odds ratio) and we assume such variables will not be important confounders. Adjusted OR and 95% CI were used to interpret the findings.

The study has got ethical clearance from the Ethical Review Committee of Jimma University. The purpose of the study was explained to the study participants and written consent was obtained. Children with malaria were treated with anti-malaria drugs based on the national guideline.

## Results

### Socio-demographic characteristics

A total of 155 mothers of cases and 155 mothers of controls were included. The mean age of the children was 30.9 (SD ± 16.51) months and males accounted for 188 (60.7%) of the total study children. A total of 141 (91.0%) mothers of cases and 123 (79.4%) mothers of controls were Oromo in ethnicity, 10 (6.5%) mothers of cases and 21 (13.5%) mothers of controls were Amhara (Table 1).

**Table 1:** Socio-demographic characteristics of respondents in Omo Nada, Tiro Afeta and Sokoru Districts, southwest Ethiopia, April, 2010.

Variable	Patient Category	
	
	Cases	Controls
Age category of the respondent		
15 - 25	49 (31.6%)	60 (38.7%)
26 - 35	94 (60.6%)	84 (54.2%)
≥36	12 (7.7%)	11 (7.1%)
Occupation of the respondent		
Farmer	76 (49.0%)	66 (42.6%)
Gov't employee	17 (11.0%)	20 (12.9%)
NGO employee	1 (0.6%)	4 (2.6%)
Student	8 (5.2%)	9 (5.8%)
House wife	49 (31.6%)	40 (25.8%)
Merchant	4 (2.6%)	16 (10.3%)
Average monthly income		
≤ 500.00	7 (4.5%)	12 (7.7%)
501.00 - 999.00	67 (43.2%)	50(32.3%)
≥ 1000	81 (52.3%)	93 (60.0%)
Educational status of the respondent		
Illiterate	77 (49.7%)	59 (38.1%)
Read and write	25 (16.1%)	25 (16.1%)
1 - 4 grade	8 (5.2%)	14 (9.0%)
5 - 8 grade	21 (13.5%)	24 (15.5%)
9 - 10 grade	13 (8.4%)	19 (12.3%)
preparatory & +	11 (7.1%)	14 (9.0%)
Family size of the household		
≤ 3	11 (7.1%)	23 (14.8%)
4-6	97 (62.6%)	96 (61.9%)
> 6	47 (30.3%)	36 (23.2%)
Type of marriage		
Polygamy	44 (35.2%)	9 (6.7%)
Monogamy	81 (64.8%)	126 (93.3%)
Number of under-five children in the house hold		
1 - 2	130 (83.9%)	136 (87.7%)
3 - 4	25 (16.1%)	19 (12.3%)
Age category of the child (in month)		
≤ 12	37 (23.8%)	34 (21.9%)
13 - 24	39 (25.2%)	36 (23.2%)
> 24	79 (50.8%)	85 (54.8%)
Sex of the child		
Male	91 (58.7%)	97 (62.6%)
Female	64 (41.3%)	58 (37.4%)

The study tried to assess the place where treatment was sought for malaria in under-five children. A total of 152 (98.1%) mothers/caretakers of cases and controls preferred health centres for under-five malaria treatment while 59 (38.1%) caretakers of the cases and 43 (27.7%) caretakers of controls preferred both health centre and health posts for the treatment of under-five malaria. The result obtained from qualitative data also showed participants preferred to visit the public health institution (Health centre, health posts) and sometimes private clinics.

### Determinants of delayed presentation

In the multivariable logistic regression analysis, five variables were independently-associated with delayed malaria treatment in under-five children. Mothers in monogamous marriage were more likely to seek treatment for under-five children late than mothers in polygamous marriage (OR = 3.41; 95% CI: 1.39-8.34). Mothers who had no history of child death were more likely to bring their under-five children late for the treatment of malaria than mothers who had history of child death (OR = 3.50, 95%CI: 1.88, 6.42). Mothers who complained about the side effects of anti-malarial drugs were more likely to have delayed under-five children for malaria treatment than mothers who did not complain about the side effects of the drugs (OR = 4.96, 95% CI: 1.21-20.36). As the cost of transportation increase, the pattern of delay increases. Mothers who lived in a village more than three kilometres from a health centre were more likely to be late for the treatment of malaria for under-five children than mothers who live less than three kilometres from the health institutions (OR = 2.01,95% CI: 1.17-3.45) (Table 2).

**Table 2:** Variables associated with delay in malaria treatment-seeking behaviour for under-five children in southwest Ethiopia, April, 2010.

Variables	Patient category		Crude OR(95% CI)	Adjusted OR(95% CI)
			
	Case	Control		
Type of marriage				
Monogamy	44 (35.2%)	9 (6.7%)	3.99 (2.08, 7.65)	3.41 (1.39, 8.34)*
Polygamy	81 (64.8%)	126 (93.3%)	1.00	1.00
History of child death				
Yes	17 (11.0%)	47 (30.3%)	1.00	1.00
No	138 (89.0%)	108 (69.7%)	3.53 (1.92, 6.50)	3.50 (1.88, 6.42)*
Presence of side effects				
Yes	40(25.8%)	4(2.6%)	13.13 (4.56, 37.75)	4.96 (1.21, 20.36)*
No	115(74.2%)	151(97.4%)	1.00	1.00
Cost of transportation				
Expensive	31(20.0%)	6 (3.9%)	12.50 (4.74, 32.94)	4.94 (1.20, 20.26)*
Optimum	58 (37.4%)	48 (31.0%)	2.92 (1.65, 5.15)	2.26 (1.11, 4.61)*
Cheap	35(22.6%)	26 (16.8%)	3.25 (1.68, 6.28)	2.69 (1.23, 5.91)*
No fee	31 (20.0%)	35 (48.4%)	1.00	1.00
Distance of health institution (m)				
≤ 3000	59(38.1%)	100(64.5%)	1.00	1.00
> 3000	96(61.9%)	55(35.5%)	2.96 (1.86, 4.70)	2.01 (1.17, 3.45)*

*P value less than 0.05

## Discussion

This study explored factors associated with delayed malaria treatment in under-five children for the first time in Ethiopia. Marital status, distances from the health institutions, and perceived side effects of the drugs were the major factors associated with delay in malaria treatment in under-five children. Occupational status of the household heads and mothers of under-five children was not associated with delay in malaria treatment for under-five children, which is in contrast with the study done in Tanzania where being a farmer was associated with delay in malaria treatment [3,6]. Similarly, educational status of mothers did not show an association with delay in diagnosis and treatment of malaria in under-five children. However, a study done in Burkina Faso showed that literacy level of the heads of the households was the main factor to bring children within 24 hours to the health facility for the treatment of malaria [21].

In this study, age and sex of the child were not associated with delay in malaria diagnosis and treatment for under-five children. Similar findings were documented elsewhere [3,6,21]. However, a study done in Nairobi slum areas showed that boys were more likely to get early diagnosis and treatment of malaria than girls [22].

Children who had monogamous parents were three times more likely to have delay in diagnosis and treatment of malaria when compared to children who had polygamous parents (OR, 3.41; 95% CI, 1.39, 8.34). Husbands in polygamous marriage may be overwhelmed by more farming activities for the livelihoods of their family. In such circumstances, decisions on health-seeking behaviour of children may be in the hands of wives who are more responsible than males for their children to seek early care. Where as in monogamous marriage, males are responsible for every decision in the household and they may be more reluctant than females to take their children to the health facility early. On the other hand, wives in polygamous marriage could compete each other to make their children healthy and alive so as to get a blessing from their husband. Polygamous family may also have good income to take sick children early to the health institution than monogamous family.

The majority of respondents have knowledge on sign and symptom of malaria as well as its prevention and control methods. However, knowledge of respondents is not associated with delay in malaria treatment. Similar studies elsewhere also showed that that knowledge on malaria transmission was not associated with timely treatment seeking behaviour for under-five children [23,24]. Knowledge about the role of mosquitoes on the transmission of malaria was not also associated with timely and appropriate help seeking behaviour for children even though such knowledge may promote personal protective measures, especially use of bed nets [23,24].

Mothers who complained about the side effect of drugs prescribed by health personnel before the illness of the current child were more likely to have delay in malaria treatment for under-five children. Due to lack of proper counselling and education, mothers may perceive that the side effects of anti-malaria drugs are very harmful than their benefits.

Mothers living within three-kilometre radius of health institution tend to seek malaria treatment for under-five children earlier. This finding supports the result of the study done in Uganda and Ethiopia which showed that mothers travelling a distance of less than three kilometres seek treatment for under-five malaria earlier than those who travel greater or equal to three kilometres [19,25]. This could be due to the location advantage of those who are nearer to the facility. In addition, the result indicated that cost incurred for transportations results in delay in malaria diagnosis and treatment.

Mothers who have history of under-five deaths tend to seek treatment earlier. This implies that perceived risk and severity of the illness affects treatment-seeking behaviours. Lack of quality control of the laboratory investigation of malaria was the major limitation of this study. As a result, there might be misclassification of cases and controls. Severity signs and symptoms of malaria among the study children were not assessed. Treatment seeking behaviour might have been affected by the severity signs of malaria.

## Conclusion

Complaint about the side effects of anti-malarial drugs, distant and cost of transportation to reach the health facilities, monogamous marriage and absence of under-five death which is a proxy measure of perceived severity of malaria were associated with delay in diagnosis and treatment of malaria. The malaria control programme should focus on increasing access to malaria treatment at the lowest facilities; address transport cost, increasing awareness of the community on early diagnosis and treatment and addressing patients concerns about the side effects of anti-malarial drugs.

## Competing interests

The authors declare that they have no competing interests.

## Authors' contributions

AG conceived the study and was involved in the analysis and Report writing. KK was involved in data analysis and review. AD was involved in the conception, design, and data analysis and drafted the manuscript. All authors read and approved the manuscript.
